# The Value of MicroRNA-155 as a Prognostic Factor for Survival in Non-Small Cell Lung Cancer: A Meta-Analysis

**DOI:** 10.1371/journal.pone.0136889

**Published:** 2015-08-31

**Authors:** Fei Wang, Jianguo Zhou, Yu Zhang, Yi Wang, Long Cheng, Yuju Bai, Hu Ma

**Affiliations:** Department of Oncology, Affiliated Hospital of Zunyi Medical University, Zunyi, Guizhou province, China; Sapporo Medical University, JAPAN

## Abstract

**Background:**

Recent studies have shown that miR-155 play a positive role in the development of carcinoma. This meta-analysis aimed to identify the role of miR-155 in the survival of non-small cell lung cancer patients.

**Methodology:**

Eligible studies were identified through database searches. Relevant data were extracted from each eligible study to assess the correlation between miR-155 expression and survival in lung carcinoma patients. The hazard ratios (HRs) and 95% confidence intervals (CIs) of the patients’ outcomes in relation to miR-155 were calculated. A total of 6 studies were included for this meta-analysis. For overall survival (OS), recurrence-free survival (RFS), disease-free survival (DFS), and cancer-specific survival (CSS), the combined HRs and 95% CIs were not statistically significant. Additionally, in Asian and America subgroups, greater expression levels of miR-155 were related to poor prognoses for lung cancer (HR 1.71 95% *CI*: 1.22–2.40, *P* = 0.002, HR 2.35 95% *CI*: 1.42–3.89 *P* = 0.001), while no significant relationship was present in a Europe subgroup (HR 0.75 95%*CI*: 0.27–2.10, *P* = 0.587).

**Conclusions:**

These results suggest that miR-155 expression is not significantly related to non-small cell lung cancer patients except in patients from Asian and America.

## Introduction

Lung cancer remains a major public health problem across the world and continues to be among the most common causes of cancer deaths in men and in women. In 2015, new lung cancer cases are expected to account for 13% and 14% of the cancer deaths in females and males, respectively. The most common causes of cancer death are cancers of the lungs and bronchi [1]. Non-small cell lung cancer (NSCLC) accounts for more than 85% of lung cancers [2]. Approximately 60% of NSCLCs are in the terminal stage. Comprehensive treatments, including surgery, chemotherapy, and radiotherapy, have been applied in lung cancer treatment, but the treatment effects are still less than satisfactory. The median overall survival for patients with NSCLC who are treated with first-line chemotherapy ranges from seven to twelve months [3]. Second- and third-line chemotherapies have been used to further increase the survival rate. Comprehensive regimes included all current therapies have been used to manage NSCLC; however, the patients’ survival rates remain unencouraging [4]. Although longer chemotherapy durations increase progression-free survival (PFS), they have had only modest effects on OS [5]. Despite the efficacy of EGFR tyrosine kinase inhibitors (TKIs), patients with EGFR mutant lung cancers eventually develop resistance to EGFR TKIs [6]. Therefore, we urgently need to identify effective treatment methods and effective therapeutic targets for NSCLC. MicroRNAs (miRs) are a class of short, evolutionarily conserved, endogenous, small non-coding RNAs that target protein-coding mRNAs at the post-transcriptional level and result in target mRNA degradation or translational inhibition [7]. miRs have been implicated in the control of many fundamental cellular and physiological processes [8]. Moreover, many observations indicate that microRNAs may be able to provide an innovative approach to the treatment of NSCLC [9–11]. It has been suggested that microRNAs may be biomarkers for cancer treatment. One of these microRNAs termed microRNA155 (miR-155) had been suggested to be closely related to the development of colorectal cancer, breast cancer and hepatocellular carcinoma following liver transplantation [12–14]. MiR-155 did predict poor survival in patients with a variety of carcinomas, but with no statistically significant in lung cancer sub-group[15]. Due to the emergence of drug resistance on the lung cancer treatment at present, exploration of a new therapeutic direction is imminent. Many studies have reported on miR-155, which might serve as a biomarker of prognoses. A pooled analysis of the currently available studies related to miR-155 in patients with advanced NSCLC was performed, and a systematic review and meta-analysis were performed to assess the prognostic value of miR-155. Also this study provided the basis of a new treatment point for later research direction. Moreover, several articles included in this review mentioned that lymph node metastases are related to miR-155 expression.

## Materials and Methods

This study was performed following the guidelines of the Preferred Reporting Items for Systematic Reviews and Meta-Analyses (PRISMA) statement (S3 Table) (http://www.prismastatement.org/statement.htm). Review protocol can be accessed on the site http://www.crd.york.ac.uk/PROSPERO/ with registration number CRD42014013428.

### Methods

Original studies that analyzed the prognostic value of miR-155 in lung cancer were identified by two participants (Yu Zhang and Long Cheng) from the PubMed, EMBASE, Web of Science, Cochrane library, CBM, CNKI and Wan Fang databases. Differences regarding study inclusion were resolved by Jianguo Zhou. The studies were selected by using the following keywords in various combinations: 'microRNA-155', 'miRNA-155', 'ir-155', 'microRNA155', 'miRNA155', 'RNA155', 'carcinoma’, ‘non-small-cell lung', 'lung neoplasms', and 'non-small cell lung cancer'. The literature published between October 2007 and October 2014 was searched. The relevant references of eligible studies were also search for additional studies to include.

### Selection criteria and Data extraction

The studies were selected and the data were extracted independently by two authors (Fei Wang and Yi Wang). A third person (Jianguo Zhou) resolved the differences and contradictions between the two authors.

The inclusion criteria were as follows: (1) the studied patients had been diagnosed with lung carcinomas (any stage or histology); (2) the expression of miR-155 in the tissue or plasma was detected; and (3) associations between miR-155 expression levels and clinical prognoses were reported, e.g., as hazard ratios (HRs), 95% *CIs* and *P* values. The exclusion criteria were the following: (1) studies related to the associations between miR-155 expression and prognosis that did not include survival analyses or lacked key information such as hazard ratio (HRs) and 95% confidence intervals (*CIs*); and (2) reviews and basic research.

The data elements of this review including the following: (1) the authors’ names, publication year, and the nationality of the studied population; (2) the characteristics of the studied population, including sample size, age, gender, tumor grade, sampling site, tumor type, and histological type; (3) miR-155 expression levels and cut-off values; and (4) HRs of for elevated miR-155 expression in terms of overall survival (OS), recurrence-free survival (RFS), cancer-specific survival (CSS) and disease-free survival (DFS) with 95% confidence intervals (*CIs*) and *P* values. If the data were not provided visually and were only provided as Kaplan–Meier curves, the data were extracted from the graphical survival plots, and estimations of the HRs were then performed using a previously described method.[16]

### Quality assessment

**Fig 1:**
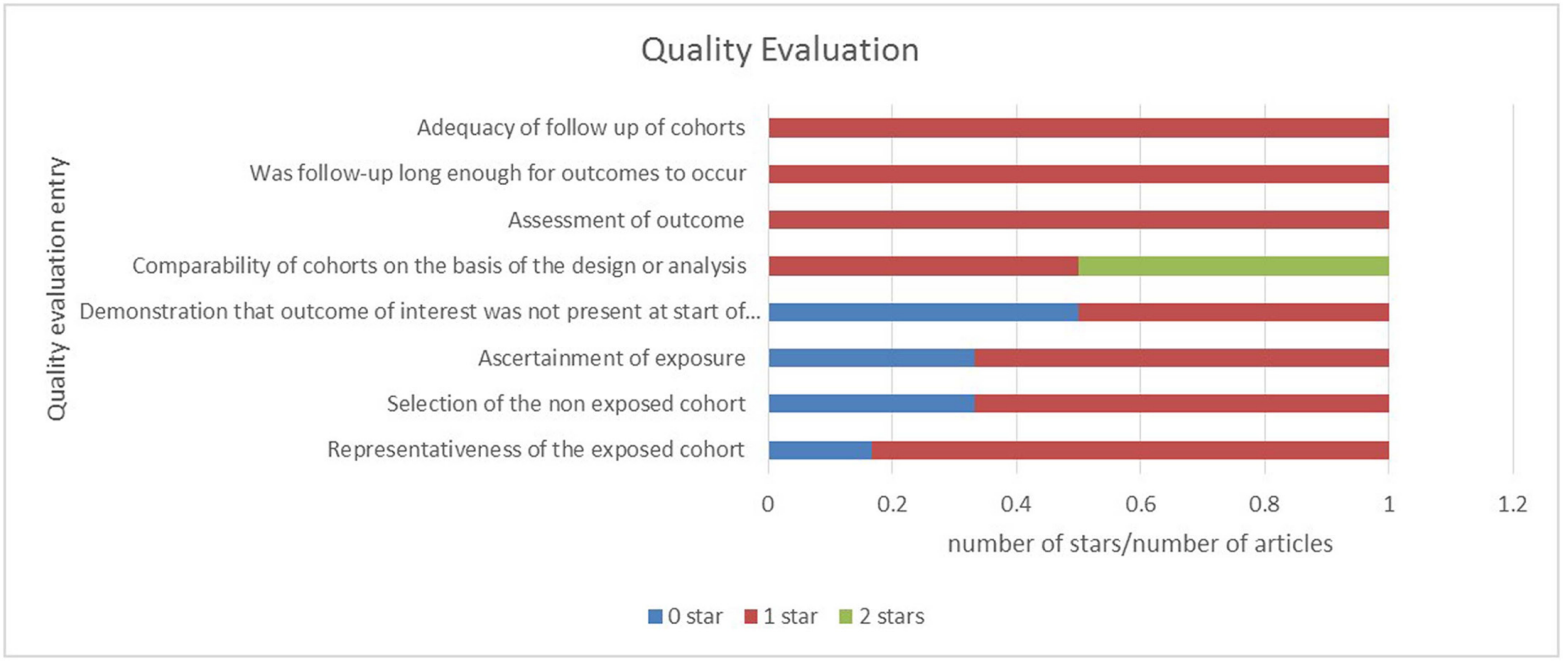
Evaluations of the qualities of the included studies based on the Newcastle-Ottawa Scale. Each band shows the percentage of the six studies with different numbers of stars.

We assessed the quality of all of the studies included with the Newcastle-Ottawa scale (NOS) for the quality of cohort studies[17]. Two investigators independently performed the quality assessments. The NOS contains three categories (selection, comparability, and outcome) and eight items. In the selection and outcome categories, a quality research item received one star, and a comparable category could receive at most two stars. In the selection part, studies that precisely described the item (i.e., those drawn from the same community as the exposed cohort with secure records and a demonstration that outcome of interest was not present at start of the study) received one star. Regarding comparability, two items (i.e., study controls as the most important factor, and study controls as an additional factor) could receive a star if the study was eligible. In the outcome portion, various items (i.e., outcome assessments, a follow-up that was sufficiently long for outcomes to occur, and adequate follow up of cohorts) could elicit one star each if the study presented the corresponding details. The quality assessment values ranged from 0 to 9 stars (S1 Table). Each band in Fig 1 indicates the percentage of the six studies that met each of these quality criteria.

### Statistical methods

The hazard ratios (HRs) and 95% confidence intervals (CIs) extracted from the eligible articles were combined for the survival results. The data were extracted from the graphical survival plots as described above. A test of heterogeneity of the combined HRs was performed using the I-squared statistic. Low, medium, and high levels of heterogeneity were delineated by I-squared values of 25%, 50%, and 75%, respectively.[18] The heterogeneity of the combined HRs would be deemed acceptable if the I-squared was < 50% (Cochrane Handbook for Systematic Reviews of Interventions Version 5.1.0, available from www.cochrane-handbook.org). A fixed effect was applied if the *P* value was > 0.05, and *P* < 0.05, resulted in the application of an analysis of the heterogeneity of the subgroups. If the subgroup analyses of multiple similar studies still revealed heterogeneity, a random effects model was used. Publication bias was evaluated with a funnel plot and the Egger’s and Begg’s bias indicator test. All calculations were performed with STATA Statistical Software Version 12.0 (Stata Corp., College Station, TX, USA) and Excel software 2013.

## Results

### Screening of the literature

**Fig 2:**
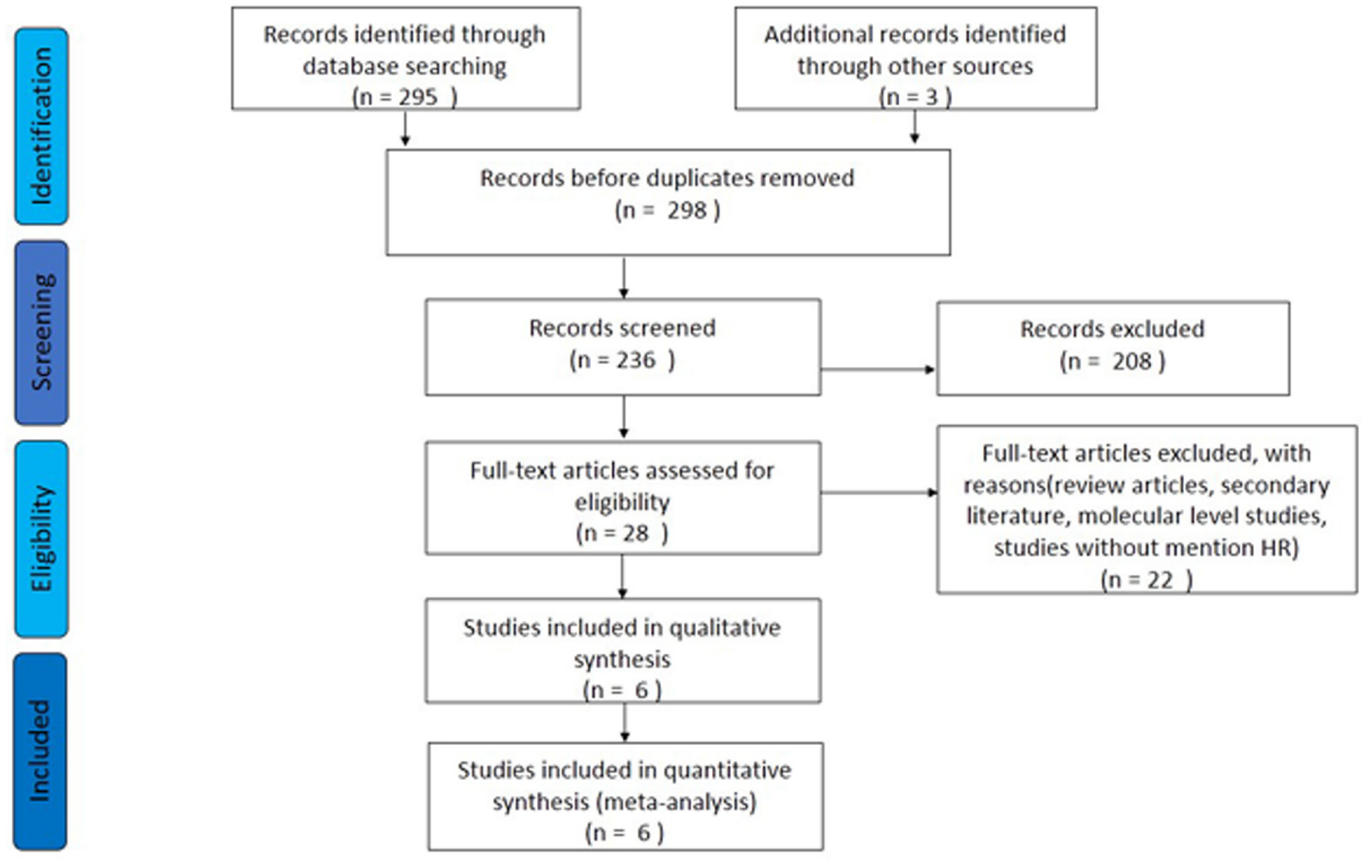
Flow chart of the study selection process.

A total of 298 studies were identified from the databases. Sixty-two were duplicates, and the remaining 236 were screened further. Based on readings of the article titles and abstracts and according to the inclusion and exclusion criteria, 28 studies were selected for further investigation. Of these 28 candidates, 5 studies were excluded for molecular research, 10 reviews were excluded, 6 studies were excluded as secondary literature, and 1 was excluded due to a lack of HRs. Therefore, 6 articles were ultimately included in the meta-analysis. A flow chart of the study selection process is shown in Fig 2.

### Characteristics of the included studies

The main features of the 6 eligible studies are summarized in S2 Table. The data collected from the 6 studies that included a total of 1557 participants were ultimately included in the meta-analysis. All of the research articles were about NSCLC. Among the articles, two used snap-frozen tissue [19, 20], two used formalin-fixed paraffin-embedded (FFPE) tissue [21, 22], one used paraffin-embedded tissue [23], and one used plasma [24]. Regarding tumor stages, one study examined stages pIIIa and pIIIb [21], two examined stages I to IIIA [23, 24], three examined stages I to IIIA [19, 20, 22]. Additionally, one study [23] used in situ hybridization (ISH) for detections, and the remaining used qRT-PCR methods. Notably, the median was selected as the cut-off value (S2 Table).

### Study outcomes

**Fig 3:**
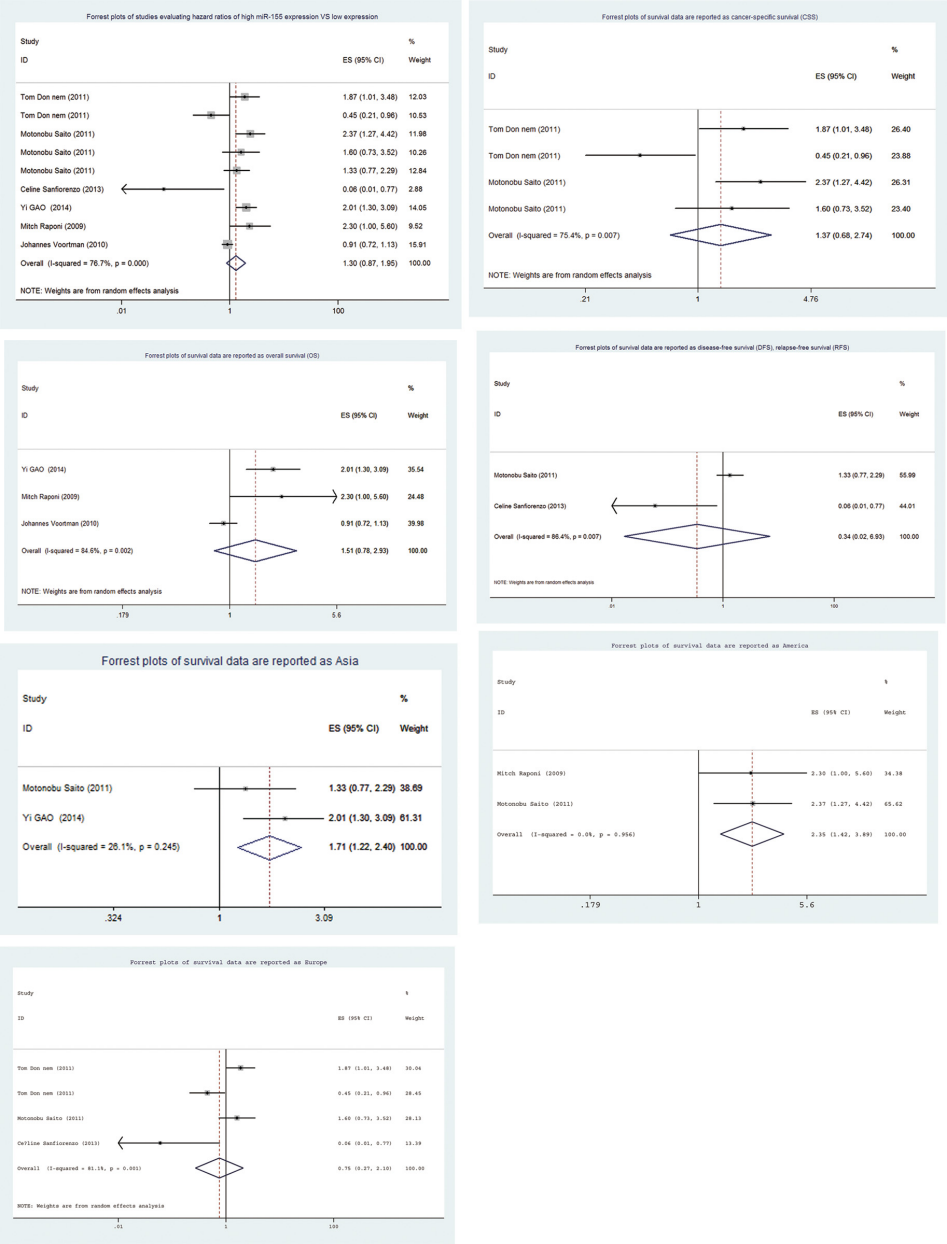
Forrest plots of the studies that evaluated the hazard ratios of high miR-155 expression vs. low expression. (A) Forrest plots of the included studies that evaluated the hazard ratios of high miR-155 expression vs. low expression. HR (95% CI) with adenocarcinoma, squamous cell carcinoma (Tom Donnem) and in Maryland, Norway, Japan (Motonobu Saito) in turn. (B) Forrest plots of the survival data reported as cancer-specific survival (CSS). (C) Forrest plots of the survival data reported as overall survival (OS). (D) Forrest plots of the survival data reported as disease-free survival (DFS) and relapse-free survival (RFS). (E) Forrest plots of the survival data from Asia. (F) Forrest plots of the survival data from the Americas. (G) Forrest plots of the survival data from Europe.

In the included articles, a close relationship between miR-155 and lung cancer prognosis was reported. However, the HRs and 95% CIs were not given explicitly in one study [21]. The HR and 95% confidence intervals extracted from the included studies were combined, and the combined results revealed that high levels of expression of miR-155 may not be related to lung cancer prognosis. After the combination of the data, a high level of large heterogeneity was observed (I-squared = 78.3% *p* = 0.000); thus, a random effects model was selected (Fig 3A). The combined HR (95% confidence interval) was 1.30 (0.87, 1.95) (*P* = 0.207).

### Survival Index

For the studies that evaluated the outcomes of CSS (Fig 3B), OS (Fig 3C) and RFS/DFS (Fig 3D) in relation to miR-155, a random model was applied because the heterogeneity between studies was large. The combined HR and 95% confidence intervals were not statistically significant (CSS: HR = 1.37, 95% *CI*: 0.68–2.74, *P* = 0.381; OS: HR = 1.51, 95% *CI*: 0.78–2.93 *P* = 0.219; RFS/DFS: HR = 0.34, 95% *CI*: 0.02–6.93, *P* = 0.483).

### Subgroup analyses

Subgroup analyses were performed according to the locations of sample collection to explore the causes of the heterogeneity between studies. The Asian and America subgroups exhibited low levels of heterogeneity (I-squared, *P* values approximately 26.1%, 0.245 and 0.0%, 0.956, HR and 95% *CI* 1.71 (1.22, 2.40) *P* = 0.002, 2.35 (1.42, 3.89) *P* = 0.001, respectively) and indicated that miR155 overexpression can lead to poor prognoses (Fig 3E and 3F). The Europe subgroup exhibited greater heterogeneity (I-squared = 81.1% *p* = 0.001), and a random effects model was employed. The HR and 95% confidence interval were 0.75 and 0.27–2.10 (*P* = 0.587, Fig 3G), respectively.

### Publication bias

**Fig 4:**
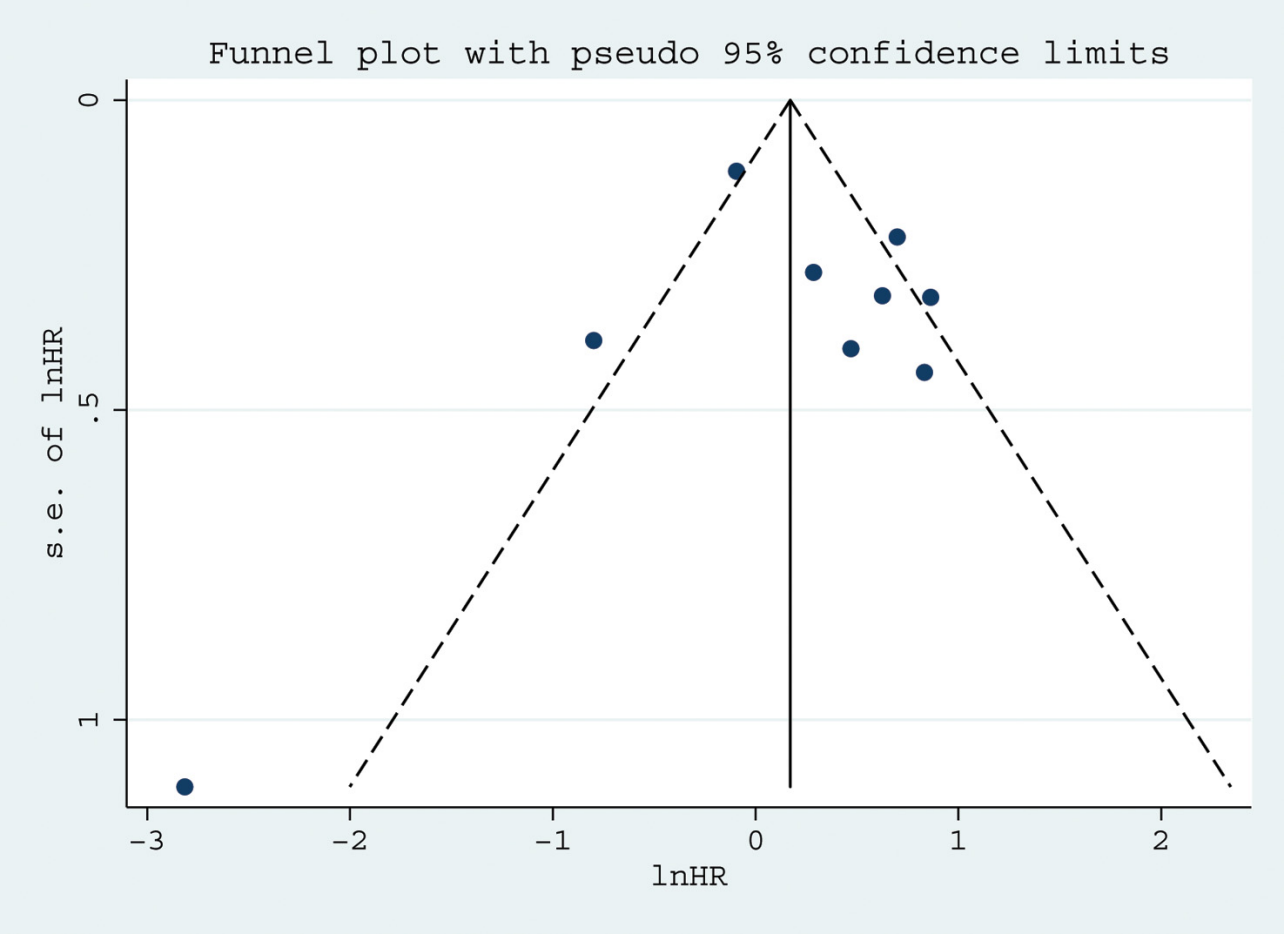
Funnel plots of studies included in the meta-analyses of NSCLC.

Funnel assay has been performed (Fig 4). Begg's and Egger's tests were used as the primary outcome indices of publication. In these tests, *P* <0.05 indicates the presence of publication bias. The tests revealed *P* values greater than 0.05 (Begg's *P* = 0.835 and Egger's *P* = 0.740) and the funnel plots were nearly symmetric thus indicated that there was no significant publication bias.

### Sensitivity Analysis

**Fig 5:**
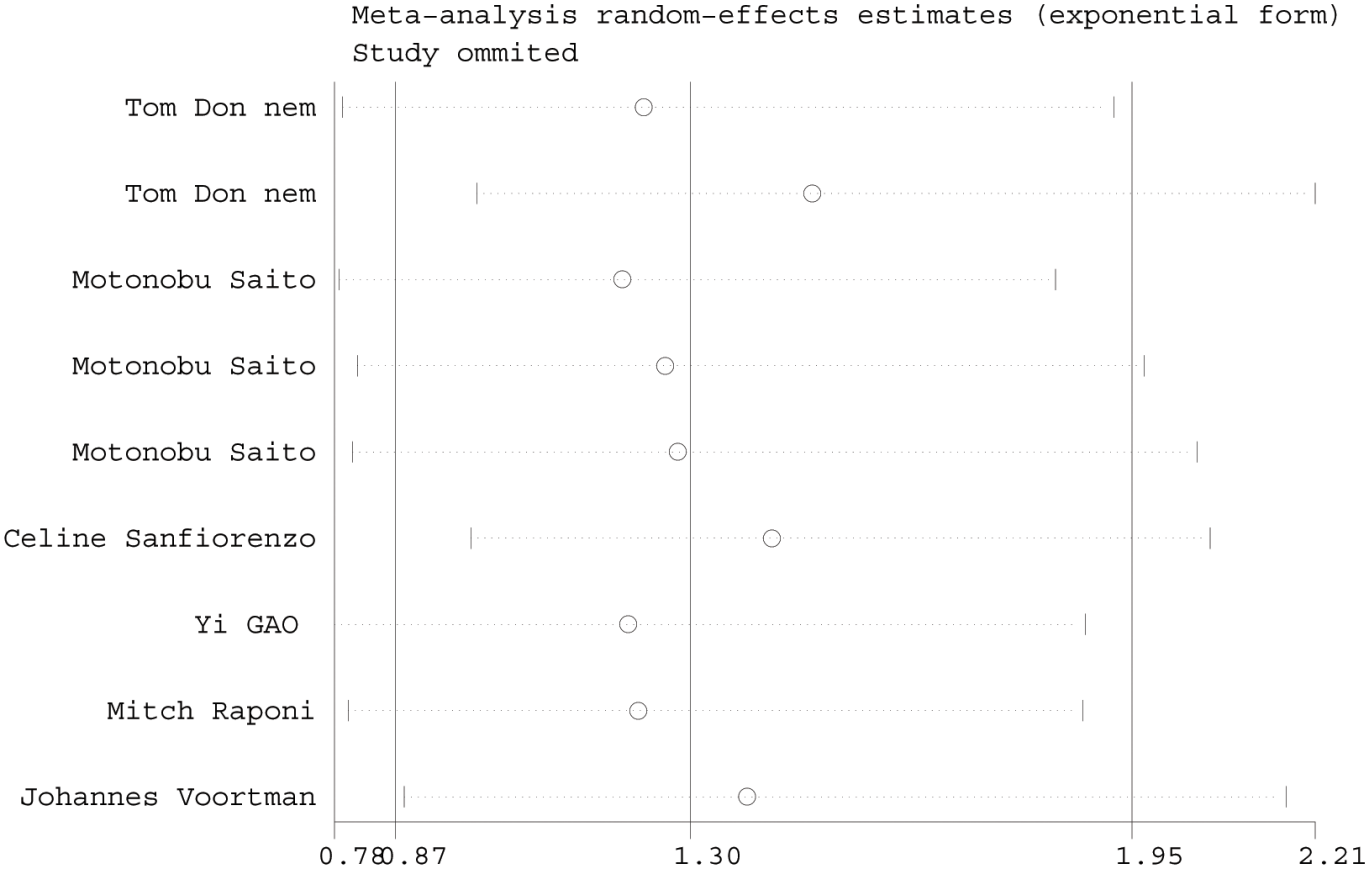
Sensitivity analysis of the HRs. The meta-analytic random-effects estimates (exponential form) were used. The results were computed by omitting each study in turn.

We also performed a sensitivity analysis to evaluate whether the differences between studies induced instability in the meta-analysis or not. The results from a random-effect model suggested the meta-analysis was stable (Fig 5).

## Discussion

A number of articles have reported that miR-155 is closely related to tumors [12, 25–27]. Cancer development may be associated with the expression of other small RNAs [28, 29], which also indicates that increased expression of miR-155 leads to poor prognoses in lung cancer. In previous study had also showed negative correlation between RFS/CSS of NSCLC and miR-155[27]. However, the conclusions from our study may be conservative. In this meta-analysis, we analyzed six articles to assess whether the lung cancer prognoses are highly associated with the expression of miR155. Study also showed the significant association with NSCLC prognosis with high heterogeneity [28]. Our study had included recently published articles to consolidate and performed the subgroup analyses to explore the causes of the heterogeneity. Regarding the OS, RFS/DFS, and CSS, the consolidated HRs and 95% confidence intervals were not statistically significant, indicating that high levels of miR-155 expression do not necessarily lead to poor prognoses in lung cancer. Further subgroup analyses based on geography revealed that when the studies from Asia and the Americas were combined, the results were significant. This new finding suggests that the overexpression miR-155 is associated with poor lung cancer prognoses in Asia and the Americas. However, in Europe, these results were not significant. Moreover, several studies have suggested that lymph node metastasis is related to miR-155 expression [3, 6, 7] and thus indicated that high levels of miR-155 expression may promote lymph node metastases.

Abnormal miR-155 expression in tumors has been associated with other aberrant RNAs (e.g., miR-146b, miR-223, and lncRNA) in some studies [30–32]. There may be interactions of other RNAs with miR-155 that cause poor prognoses in NSCLC and thus may have induced this this outcome. A previous study examined the relationship between miR-155 and lung cancer, and the OS data were found to be non-significant [33]. We included newly published studies and performed further subgroup analyses according to country. The distributions of the relevant populations may also be relevant to this conclusion. Moreover, the poor qualities of some of the included studies and the small number of eligible studies cannot be ignored, and these factors may have affected the results of studies. The sampling sites utilized in the include studies can be divided into plasma and tissue. The standard deviations of miRNA expression in these two sampling sites are different, and this difference may have contributed to this conclusion. We did not perform a subgroup analysis according to sampling site because only a single study dealt with plasma. The presence of heterogeneity among the eligible articles and other factors, including in publication bias and the different research methods of each article, also potentially influenced the results. The significant heterogeneities in the subgroup analyses in terms of OS, RFS/DFS, and CSS may have been caused by differences in baseline characteristics, including the number of patients, sex, age, tumor stage, and follow-up time.

## Conclusions

In summary, this meta-analysis revealed that higher miR-155 expression levels were partially associated with poor survival in patients with lung cancer. However, further study is required to resolve the questions of whether miR-155 can serve as a prognostic biomarker and whether it can be used as a biomarker of lymph node metastasis.

### Appendix: PubMed search terms

#1 Search (Carcinoma, Non-Small-Cell Lung[Me SH Terms]) OR Lung Neoplasms[Me SH Terms]

#2 Search (non-small cell lung cancer[Title/Abstract]) OR lung cancer[Title/Abstract]

#3 Search (((Carcinoma, Non-Small-Cell Lung[Me SH Terms]) OR Lung Neoplasms[Me SH Terms])) OR ((non-small cell lung cancer[Title/Abstract]) OR lung cancer[Title/Abstract])

#4 Search (((((microRNA-155[Title/Abstract]) OR microRNA155[Title/Abstract]) OR miRNA155[Title/Abstract]) OR RNA155[Title/Abstract]) OR RNA-155[Title/Abstract]) OR miRNA-155[Title/Abstract]

#5 #3 and #4

## Supporting Information

S1 Table Evaluations of the qualities of the included studies based on the Newcastle-Ottawa Scale. (DOCX)

S2 Table Characteristics of the included studies. (DOCX)

S3 Table PRISMA 2009 Checklist. (DOC)
