# Supplementary material for: The Value of MicroRNA-155 as a Prognostic Factor for Survival in Non-Small Cell Lung Cancer: A Meta-Analysis
Source: PLoS One. 2015 Aug 31;10(8):e0136889. doi: 10.1371/journal.pone.0136889 (PMC4556438; doi:10.1371/journal.pone.0136889)
Supplement: S1 Table — (DOCX) [file pone.0136889.s001.docx]

(S1 Table) Evaluations of the qualities of the included studies based on the Newcastle-Ottawa Scale

|  | **Selection** |  |  |  |  | **Comparability** |  | **Outcome** |  |  |  | **total** |
| --- | --- | --- | --- | --- | --- | --- | --- | --- | --- | --- | --- | --- |
| Author | 1)Representativeness of the exposed cohort | 2) Selection of the non-exposed cohort | 3) Ascertainment of exposure | 4) Demonstration that outcome of interest was not present at start of study |  | 1) Comparability of cohorts on the basis of the design or analysis |  | 1) Assessment of outcome | 2) Was follow-up long enough for outcomes to occur | 3) Adequacy of follow up of cohorts |  |  |
| Yi Gao (2014) | * | 0 | * | 0 |  | * |  | * | * | * |  | 6 |
| Mitch Raponi (2009) | 0 | * | 0 | 0 |  | ** |  | * | * | * |  | 6 |
| Motonobu Saito (2011) | * | * | * | * |  | * |  | * | * | * |  | 8 |
| Tom Don nem (2011) | * | * | 0 | * |  | ** |  | * | * | * |  | 8 |
| Johannes Voortman (2010) | * | * | * | * |  | ** |  | * | * | * |  | 9 |
| CelineSanfiorenzo (2013) | * | 0 | * | 0 |  | * |  | * | * | * |  | 6 |

In the selection and outcome categories, a quality research item following (studies that precisely described or eligible) received one star, and a comparable category could receive at most two stars. The quality assessment values ranged from 0 to 9 stars.
