# Supplementary material for: The Value of MicroRNA-155 as a Prognostic Factor for Survival in Non-Small Cell Lung Cancer: A Meta-Analysis
Source: PLoS One. 2015 Aug 31;10(8):e0136889. doi: 10.1371/journal.pone.0136889 (PMC4556438; doi:10.1371/journal.pone.0136889)
Supplement: S2 Table — (DOCX) [file pone.0136889.s002.docx]

(S2 Table) Characteristics of the included studies

| Author | Country | Enrolled | Tumor grade (I.II/III.IV) | Sampling site | Tumor type | Histological type | Method | Follow-up time (median)month | cut-off | Survival outcome | HR (95% CI) | P | Lymphatic invasion | survival analysis (month) |
| --- | --- | --- | --- | --- | --- | --- | --- | --- | --- | --- | --- | --- | --- | --- |
| Yi GAO (2014) | china | 162 | pIIIa, pIIIb | FFPE | NSCLC | ADC SCC | qRT-PCR | 14.5 | median | HR | 2.01 (1.3-3.09) | NG | P=0.032 | OS |
| Tom Donnem (2011) | Northern Norway | 335 | I to IIIA | PET | NSCLC | ADC SCC | ISH | 86 | median | HR | In ADC 1.87  (1.01 - 3.48) In SCC 0.45 (0.21-0.96) | 0.047  0.039 | P=0.034 | CSS |
| Motonobu Saito (2011) | Maryland  Norway  Japan | 89 37  191 | I to III | SFT | NSCLC | ADC | qRT-PCR | 80 | median | HR | 2.37 (1.27–4.42)  1.60 (0.73–3.52)  1.33 (0.77–2.29) | 0.006  0.245 0.309 | NG | CSS  CSS  RFS |
| Mitch Raponi (2009) | USA | 54 | I to III | SFT | NSCLC | SCC | qRT-PCR | 36 | median | HR | 2.3 (1.0–5.6) | 0.06 | NG | OS |
| Johannes Voortman(2010) | 28 centers in 14 countries | 637 | I to III | FFPE | NSCLC | ADC SCC | qRT-PCR | 96 | median | HR | 0.91 (0.72–1.13) | NG | 0.001 | OS |
| Ce´line Sanfiorenzo (2013) | France | 52 | I to IIIA | plasma | NSCLC | ADC SCC | qRT-PCR | minimum 18 | median | HR | 0.060 (0.005–0.767) | 0.03 | NG | DFS |

Abbreviations: OS, overall survival; RFS, recurrence-free survival; CSS, cancer-specific survival; DFS, disease-free survival; FFPE, formalin-fixed, paraffin embedded; PET, paraffin-embedded tissue; SFT, Snap-frozen tissue; ISH, in situ hybridization; ADC, adenocarcinoma; SCC, squamous cell carcinoma; NSCLC, non-small cell lung cancer; qRT-PCR, quantitative real-time polymerase chain reaction; NG, not given.
